# Genre Complexes in Popular Music

**DOI:** 10.1371/journal.pone.0155471

**Published:** 2016-05-20

**Authors:** Daniel Silver, Monica Lee, C. Clayton Childress

**Affiliations:** 1 Department of Sociology, University of Toronto Scarborough, Toronto, Ontario, Canada; 2 Department of Sociology, University of Chicago, Chicago, Illinois, United States of America; University of Maribor, SLOVENIA

## Abstract

Recent work in the sociology of music suggests a declining importance of genre categories. Yet other work in this research stream and in the sociology of classification argues for the continued prevalence of genres as a meaningful tool through which creators, critics and consumers focus their attention in the topology of available works. Building from work in the study of categories and categorization we examine how boundary strength and internal differentiation structure the genre pairings of some 3 million musicians and groups. Using a range of network-based and statistical techniques, we uncover three musical “complexes,” which are collectively constituted by 16 smaller genre communities. Our analysis shows that the musical universe is not monolithically organized but rather composed of multiple worlds that are differently structured—i.e., uncentered, single-centered, and multi-centered.

## Introduction

How do popular artists form their public identities by mobilizing existing stylistic forms? Strong evidence suggests that “categorical imperatives” [1] impose penalties on producers for illegitimate role performance, especially when performance is evaluated by critics and discriminating audiences, as it is in the music industry. Much research, moreover, argues that musical genre expectations in particular profoundly organize the music industry [2–4], shaping how band members meet [5–6], producers choose and venues book bands [7–8], radio stations choose what to play [9–10], record label divisions are organized, music news is reported, as well as how fans find music to enjoy and people to enjoy it with [4]. As such, genre designations and expectations provide crucial reference points that inform the way musicians construct their public presentation of self.

At the same time, other research indicates that (some) genre expectations are weakening [11–12], being more flexibly reimagined [13], being redefined as search algorithms (such as Pandora’s Music Genome Project) that create new ways to sort music independent of style, and even fading away in some contexts to the point that major digital musical stores like iTunes barely mention genre [10]. These transformations may in turn reconfigure the traditional genre frameworks through which musicians present themselves to their various audiences; rather than being a fixed and static system, genres emerge, evolve, and change over time [5].

Building on this research, we examine the structure of genre self-classification by popular musicians. We propose that big data sources such as MySpace.com make it possible to empirically and comprehensively evaluate debates about the strength and types of genre classification at work in popular music. Specifically, we ask:

- Which, if any, genre conventions structure popular music?
- How does the strength and structure of genre conventions differ across musical styles?

We pursue these questions in the context of two related literatures, one on music specifically and the other on the sociology of classification more generally.

We start in the sociology of music, where a pressing question concerns the extent to which traditional genre categories continue to structure the social production of music in the face of various pressures toward more flexible modes of categorization. We join this literature with ideas from the sociology of classifications. This literature helps us to move beyond the binary question of whether categories are strengthening or weakening to more fruitful questions about how various boundary characteristics, such as their clarity or scope, interrelate.

Following DiMaggio [14], we suggest *ritual strength* and *differentiation* as key dimensions for cultural classification, and treat these as different but complementary aspects of genre classification. Crossing these two dimensions produces a four-fold typology of what we call “genre complexes”: *multi-centered*, *uncentered*, *single-centered*, and *free interchangeability*. Our analysis of some 3 million musician profiles on MySpace.com indicates that Rock musicians categorize themselves in a *multi-centered* way, Hip Hop musicians in a *single-centered* way, and musicians in non-commercial or “niche” genres in an *uncentered* way; free-interchangeability, although theoretically possible, was not present in our data.

The primary goals of this paper are thus three-fold. A first contribution is *theoretical-synthetic*: we review existing literature on both genre and classificatory structure more broadly and sort key positions in the field into a more generalizable typology of theoretically possible intersections of two simple principles, *differentiation* and *boundary strength*. A second contribution is *methodological*: we demonstrate how to use network-analytical techniques to discern aggregate patterns of individual musicians’ genre choices from big data sources. A third contribution is *empirical*: we report on the structure of MySpace.com users’ genre self-presentation as of 2007 and show that the resultant patterns closely approximate fields within our theoretically derived typology. While much sociological work examines genre boundaries as they intersect with industry imperatives and audience demand, we investigate musical genres as a self-contained relational system and aim to illuminate its structural principles and aggregate patterns. We conclude with suggestions for extending this research further, in particular by examining how characteristics of musicians’ genre categorization relate to their popularity and their surrounding social contexts.

## Genres in the System of Music Production

### Musicological vs. Sociological Conceptions of Music Genre

Literary and music scholars often treat genres as common stylistic elements that differentiate classes of artworks from each other. Thus imaginative writings in the romance novel genre exhibit certain key character-types, plot developments, and story structures [15]. Similarly, musics exhibit generic forms of melody, lyric, mood, rhythm, harmony, and instrumentation. Music genre in this conception is a musicological category discerned by analyzing the musical and lyrical contents and structures internal to musical works [16, 3].

Sociologists by contrast have stressed genre as a system of social classification [14]. Sorting people and things into categories is a crucial social process [17–20], defining reference groups and appropriate behaviors while channeling gatekeeper and consumer attention in specific directions that may favor or hurt the chances of success for products and performers [1, 21–25]. Musical genres in particular provide a set of shared expectations (about music and sometimes life more generally), which deeply structure musical production and consumption [3, 10, 26]: collaboration among musicians, music media writing, radio airplay, concert listings, record label marketing and talent acquisition, and more [3, 4, 9, 10, 27].

On this sociological view, genres are not so much common musicological elements as typical forms of interactions based on normative expectations. Hence Lena [3] discerns four major genre forms among American popular musical styles: avant-garde, scene-based, industry-based, and traditionalist. The difference between these four lies in the social dimensions that differentiate musical styles, such as organizational form, organizational scale, or the function of typical dress and argot. Musics classified within a given genre form are subject to different normative expectations and conflicts: musicians working within scene-based genres are expected to sustain local communities organized around their music, and face sanctions for producing work for the mass market; musicians working within industry-based genres are expected to sell records, and face sanctions for reducing their marketability.

Two major conclusions flow from this sociological intervention in the analysis of music genre. First, music genres are not wholly defined by their sonic qualities. Second, the non-sonic qualities of music genres often emerge relationally and categorically: relationally, because different musicological genres can share structural similarities and differences with sonically “unrelated” genres; categorically, because any given genre acquires some part of its meaning in virtue of its position within a broad and diverse topology of higher-order genre forms.

### Genre Boundaries Might Be Weakening

A stream of research in the sociological literature proposes several mechanisms that may be fundamentally altering the way traditional boundary categories operate. The central process is a version of Durkheim’s division of labor thesis, which we could re-formulate in cultural sociological terms as DiMaggio’s [14] “proposition C-6”: “the more differentiated the system of genre classification, the less universal.” Or, in a statement by Lester Bangs quoted by Bruce Springsteen at his South by Southwest keynote speech: “Elvis was probably the last thing we were all going to agree on” [28].

We can spell out this logic in more detail. Less differentiated fields tend to be marked by more total oppositions that demand more wholehearted commitment. As specialization advances, new combinations become possible. In the realm of musical genres an example would be rock music differentiating into “hard” subgenres such as hard-core and metal while at the same time “rap” spins out its own hard-core variants, thereby creating the possibility of metal-rap crossover through the hardcore sub-genres of each genre. Such a hybrid would have seemed “impure” if only larger, homogenous classifications such as rap and rock were available. But with finer-grained classifications, new categories that need not respect old boundaries become possible. Combinations between seemingly unrelated sub-genres such as rap-folk or Nintendo Core should become more likely to occur, along with other novel combinations such as pop-punk or Avant-garde metal. By extension, as genre categories approach infinity, their capacity to constrain behavior would approach zero and in effect genre classification itself would become socially meaningless.

Differentiation, as Durkheim observed, is often driven by increasing “dynamic density” in a field. This occurs when producers are brought into closer communication, stimulating competition, enabling collaboration, and expanding their reference points for evaluating themselves. In the latter-half of the twentieth century, key drivers of genre differentiation include domestic and international migration, new communication technologies, legal frameworks, inter-organizational competition [26, 29–31], and technologies that permitted increased competition among radio stations and the targeting narrower market niches [32–33].

More recently digital technologies have promised to rapidly and deeply transform –and ultimately weaken –systems of musical genre classification [34–37]. Several mechanisms may be at work. Music scenes are no longer restricted to a specific physical locality. A band’s social media profile can be viewed anywhere in the world, making it possible for musicians working anywhere to know about, influence, and remix each other’s work, regardless of genre or sub-genre [35]. Online music stores are essentially unlimited in size and their products can be categorized in infinite ways. This makes it both harder for consumers to sift through the various offerings and possible to generate more flexible classifications sensitive to consumer preferences and behavior rather than pre-defined genre categories. Algorithms like the ones used by iTunes, Amazon, and Pandora strive to predict consumers’ musical preferences based not on genres but on past choices of similar consumers or analogies between a purchased song and other songs. Genres thus fade in consumer salience. Similarly, social networking websites (like Facebook or Twitter) may increase the salience of network ties in music diffusion while decreasing the salience of genre labels and other traditional forms of classification [10]. Instead of looking to genre classifications to provide information about how to program or advertise a new song, industry professionals may instead look to social network information. One might accordingly imagine a world in which music sales are sorted not into charts based on generic categories but instead based on user-specific contexts (top songs for people in my Facebook friend, friend-of-friend, etc. network).

This line of research implies at least two general hypotheses about the overall trajectory of the popular music system: 1) an increasing *complexity* of genre categorizations over time, and 2) as genre-based boundaries of classification systems break down, genres are replaced as signaling mechanisms by alternative social indicators of taste and preference. Or, put another way, some recent work in sociology—not to mention Lester Bangs and Bruce Springsteen—(implicitly) point toward a hypothesis about the contemporary structure of popular music genres: as genre boundaries become more fully porous, and genre as a signaling device to audiences and industries grows increasingly unimportant, there should be large and growing domains of *free interchangeability* in musicians’ selection of genre combinations in defining their own work.

### Genre Boundaries Should Nevertheless Persist

At the same time, the sociological literature gives us strong reason to believe that genre categories should persist in structuring musical production and consumption. Consider just a few possible reasons for this persistence.

Producers are likely to rely on generic categories, even if consumers and critics have less need to do so. Record company executives and radio programmers have to allocate scarce resources. Even Internet radio has to allocate time and attention. Such gate-keeping decisions are likely to rely on genre categories [38], even while online *consumers* presented with customized streams of information may have less need for genre categories in their decisions [10].

Musicians are also likely to rely on genre categories for finding collaborators. Again unlike iTunes shoppers, musicians do not necessarily select collaborators based on flowing information streams. They look for others (musicians, producers, agents, venues) with whom they can work and from whom they can learn. Genre classifications send strong signals in this regard, helping musicians to sort one another into those with whom they might collaborate or not. For instance, musicians in a scene-based genre may be hesitant to work with representatives from an industry-based genre [3].

Musicians moreover may rely on genre categories for self-advertising. To the extent that music industry gate-keepers and scene members continue to use genre categories to make decisions, musicians aspiring to commercial success or scene-acceptance will feel pressure to do so as well. For instance, if country music airplay and commercial marketing strongly depends on conformity to genre conventions (of dress, speech, lyrical content, and even political orientation), then self-identifying as a “country” musician will be an important and expected professional statement by a musician aspiring to commercial success in that field [5, 10].

Even beyond showing allegiance to genre-specific codes, genres, as a form of categories more generally, may also serve as strong incentives in connecting with audiences. Thus the sociology of classification has long been preoccupied with the fuzziness or clarity of the boundaries between categories. Fuzziness or clarity, according to this literature, is a function of how strongly those boundaries are demarcated. Much of this work examines how producers fare in a market where genres/categories are more or less clear [21, 24, 39], finding that attention and interest impose powerful incentives on maintaining clear categorization. Other work, by contrast, highlights categorical diversity, noting that a market is made up of multiple genres or categories, and producers face the challenge of situating themselves within or across this multiplicity [24, 40].

While for creators the maintenance and clarity of genre categories may be a device to attract attention and remuneration from audiences, for their part, music fans may continue to rely on genre classifications for identity formation and inter-personal relationships. Music consumption goes beyond buying songs on iTunes. Music “fandom” often involves ethical convictions, political attitudes, styles of sociability, manners, race privilege and protest, and more [6, 41–45]. To the extent that these are encoded in genre categories, such labels should be relatively sticky forms of social classification.

This line of research thus implies the following general proposition: despite the increasing complexity of genre designation and increased reliance on extra-genre sorting mechanisms, genres should nevertheless persist as significant methods of sorting and sense-making in musical spheres.

### Multiplicity and Complexity

The above discussion highlights research focused on the question of whether traditional genre classifications are strengthening or weakening, and at the extremes whether there is reason to believe they will continue to exist at all. Yet there need not be one monolithic pattern describing the current state of genre boundaries in the overall system of popular music. Some regions within that system may have weak boundaries, others strong; some may be highly differentiated, others relatively undifferentiated. Any given genre is part of a larger complex or world, whether understood as a stream [46], social form [3], or otherwise. These worlds exert distinctive types of normative pressures, which have implications for how musicians are expected to relate to genre boundaries. Thus, genres likely exhibit different structural tendencies, depending on how they coalesce into larger complexes. For instance, some complexes may promote sub-cultural identity and novel mixing while others may encourage integration, expansion, and commonality [11, 45]. The more general literature on the sociology of classifications helps to articulate this multi-dimensional possibility.

Although much work on classification systems focus on single dimensions of classification (such as fuzziness-clarity or diversity-homogeny), this is not true of all work within this stream. Clarity and multiplicity, for instance, are two separate dimensions that together define four possible types of categorical schemas. There may be a small number of categories with clear boundaries; there may be a large number of categories with fuzzy boundaries, and so on. For instance, Kovacs and Hannan’s [47] examination of audience ratings of restaurants in San Francisco considers the effects of combining different levels of boundary clarity and categorical diversity. Restaurants may remain within one category, they may span multiple fuzzy categories, or they may span multiple distinct categories. Kovacs and Hannah [47] find that when categories are low contrast, there are relatively few penalties for spanning them; when categories are high contrast, spanning them incurs harsher penalties. Likewise, Negro et. al’s [48] examination of winemakers finds that specialists have a big advantage in high contrast conditions but less advantage in low contrast conditions.

These works incorporate research on both boundary strength and categorical diversity, a trend that is in large part a rediscovery within a different research domain of DiMaggio’s study of artistic classification systems [14]. DiMaggio proposed that artistic classifications vary according to four dimensions—differentiation, hierarchy, universality and ritual strength. Differentiation—the number of genres into which a classification system is divided—and ritual strength—the intensity with which genre boundaries are defended in artistic production and consumption—closely resemble the concepts of categorical diversity and boundary strength that are central to the literature cultural categorization. Classification systems may be examined empirically according to any or all of these dimensions.

### A Typology of Musical Worlds

This line of inquiry suggests treating genre systems not one-dimensionally but as *complexes* of different but compatible subsystems that cohere in what might be termed “musical worlds.” Given that *boundary strength* and *differentiation* have been key themes both in the recent literature and in DiMaggio’s [14] classic formulation, we take their intersections as a theoretical starting point for our empirical analysis of how musicians in fact tend to combine genres. Some genre complexes may have very strong boundaries, rarely intermingling with other styles; some less so. Some are internally differentiated, breaking down into sub-genres, while others do not break down in such an organized manner. As a result, arguments for and against the prevailing importance of genre differentiation may not apply to the entire musical landscape, but may instead reflect some “musical worlds” and not others within the overall topography of music. Accordingly, we construct a 2x2 table showing how genre complexes may differ according to their boundary strength or internal differentiation.

Table 1 shows four ways genre complexes may be organized in terms of the intersection of boundary strength and internal differentiations. High strength and high differentiation describes a multi-centered system in which distinct subcultures interpenetrate. High strength and low differentiation describes a single-centered complex, where, within a strong external boundary, genres fluidly mix in an unpatterned way. Low strength with high differentiation describes unbounded and uncentered space of subcultural mixing; multiple constellations that do not stay within a given galaxy.

**Table 1:** A typology of musical worlds.

	High Differentiation	Low Differentiation
**High Boundary Strength**	*Multi-centered*: bounded subcultural interpenetration	*Single-centered*: bounded fluidity
**Low Boundary Strength**	*Uncentered*: unbound subcultural mixing	*Free interchangeability*: unbound fluidity

Our analysis as it unfolds below gives more substance to this typology of genre systems. To preview our main finding: MySpace musicians classify themselves according to three major complexes of genres—one composed of Rock music genres, one of Hip Hop genres, and one of Niche or non-commercial genres. These complexes, as we will see, define three major “musical worlds,” and exhibit divergent structural patterns.

## Data

### MySpace.com: A Window onto Popular Music

To discern the structure of the genre complexes into which musicians sort themselves, we employ a powerful dataset: musician profiles on MySpace.com, downloaded in January, 2007. MySpace.com is an internationally known website popular for its social networking capabilities, and used heavily by musicians seeking to promote their work [49]. Since 2007 other services like facebook.com have also become popular among musicians, but as of 2007 MySpace.com was the most widely-used and lacked serious competition. At that time, it received even more traffic than google.com. MySpace.com thus provides an extremely powerful window into the activities of popular musicians.

Although the use of data derived from social networking sites is a relatively new phenomenon, there is considerable literature that utilizes the MySpace.com website as a primary data source [50–59]. All have included smaller sample sizes than our study: across the nine articles just cited, N’s ranged between 1 MySpace user profile and 1.9 million user profiles, with most (6 of 9) including less than 30,000 MySpace user profiles. Our database includes nearly 3 million profiles: all musician profiles that were available for public viewing.

As have other researchers, we use data acquired through a software tool that scans the MySpace site and extracts user profile information for further analysis. Our data were initially gathered by the University of Chicago Cultural Policy Center with a custom algorithm, and was first used in Rothfield et al. [60] (see p. 50, where details about the script and data gathering methods can be found). All data were collected and used according to the company’s Terms and Conditions. Prior research using this dataset has examined descriptive features such as city-by-city genre distributions [61].

**Fig 1:**
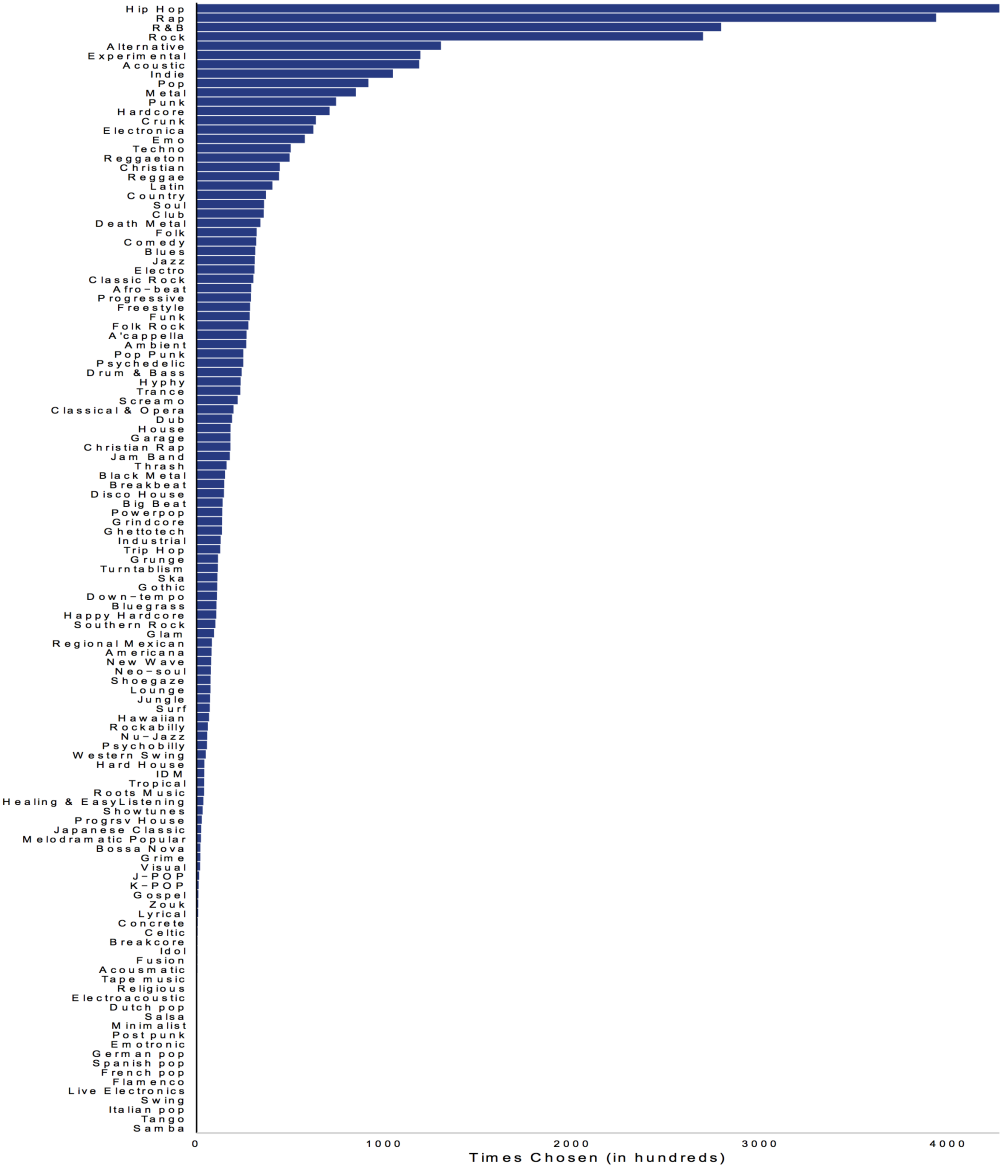
Frequency of Genre Selection.

A key feature of MySpace profiles makes them especially useful for our purposes. Musicians self-selected genres for their profiles; their profiles thus record choices about how musicians deploy genres in their self-presentations. They did so from a list of 122 categories: a large but manageable number, especially for self-identified musicians who likely are more keenly aware of subtle connotations of genre labels than the general populace. Moreover, musicians could choose up to three genres. This means that if they wished to do so, musicians could combine genres in conventional ways (e.g. Rap, Hip-Hop, R&B) or unconventional ways (e.g. Showtunes-Americana-Death Metal). Such genre combinations constitute the core of our analysis, below, as they allow us to observe the clusters into to which musicians tend to consistently combine genres.

(Fig 1) shows basic descriptive statistics for the genres included in the MySpace dataset. It ranks the 122 MySpace genres by how frequently bands select them. Rap, Hip Hop, R&B and Rock, are the most commonly chosen; Samba, Tango, Italian Pop, and Swing, are the least common.

To be sure, this data set has its limitations. Online identities are malleable [62–64]. People may brag, file incorrect information as a joke, lie to increase their status, and the like. Many MySpace profiles may not meet normal social standards for being a “band” or “musician” (e.g. they may not be public or popular enough). Even though we have a large enough N to claim some degree of generalizability for this study’s results, certainly not all musicians have a MySpace page. Moreover, the available genres on MySpace may influence musicians’ choice patterns.

Despite these limitations, there are considerable benefits to the Myspace data over previously available datasets. First, it is one of the largest available datasets of currently active (at the time of 2007) popular musicians. That MySpace users do not include all musicians likely means that the dataset is somewhat biased away from older and more traditional musicians and toward younger, more digitally-savvy musicians. This means that all of the processes associated with the Internet reviewed above should be especially salient in this group, making it all the more meaningful that we find (below) strong genre boundaries even here. Second, whereas these data are broad in their definition of “band” or “musician” through the use of self-selection into the category, they are freed from the limitations of extreme censoring of “small”, “marginal”, “upstart”, or generally “unknown” bands through other collection strategies. In addition, these data provide a powerful and relatively unique opportunity to examine bands who have *self-defined* their music along genre indices for public appraisal and consumption. As such, due to both our large N and the richness of the data we are able to treat atypical genre combinations as *useful information* rather than “error” or outliers; they may indicate that (some) musicians do not feel bound to respect standard or traditional genre combinations and conventions, and perhaps may even seek unconventionality. To examine the exceptions, however, we must first discern the rule, and this is the major goal of the present paper. Finally, while different data sources collected at different times may reveal different patterns from those we find in MySpace, in order to develop and test coherent hypotheses about the nature and direction of such differences, we need revenant theoretical frameworks, technical methods, and empirical reference points, which this paper seeks to provide.

## Analysis

### Analytical Strategy

Do musicians choose genres in regular patterns that form discrete complexes or are their choices relatively unbounded? If they are bounded, what holds them together? How do they vary in terms of their boundary strength and their internal differentiation? These questions guide our analysis of the structure of genres in the MySpace musical universe.

We follow a three-stage strategy in analyzing the MySpace data. Methods are discussed in the course of the analysis. A *first* step is to reject the null hypothesis: that there is a completely random relationship among a band’s genre choices. Whatever a band chooses for genre 1 would be arbitrarily related to its choice for genre 2 and genre 3, and vice versa. This is admittedly an unlikely scenario, but it does provide a useful baseline while simultaneously doubling as a test of whether the *free interchangeability* hypothesis holds as a system-wide phenomenon, in which genre assignments are combined seemingly at random.

Using a community detection (modularity optimization) algorithm, we find that genre choices are far from random. Certain genres are paired with one another with great consistency. To demonstrate this, we catalogue all musician-supplied genre combinations as a network defined by the frequency with which bands co-select them. For instance, if one band chooses Rap and Metal, there would be one edge between the “Rap” and “Metal” nodes, and so on with numerical frequency for “Rap” and “Metal” and all other activated genre-by-genre ties.

Given that bands’ genre choices do evince latent structural patterns that are not captured by modularity, the next (*second*) question is to examine the nature of these patterns. By applying modularity clustering to the MySpace genre hierarchically—that is, repeatedly subdividing genre communities until it is impossible to do so again with statistical significance—we are able to characterize in greater detail the MySpace universe’s organization of musical genres. We find a fundamental first division among Rock, Hip-Hop, and Niche/non-commercial musical worlds that break down further into 16 distinct genre communities.

Given that we do find that MySpace musicians group genres into consistent complexes (3 worlds, 16 genre communities), we turn to our *third* question, about the structural dimensions of these complexes. To do so, we examine the extent to which the permeability of genre communities’ boundaries vary. Here we return to the 2x2 table from above, and show how the major genre complexes in the MySpace universe fit within it.

### Musicians Combine Genres in Strongly Patterned Ways

MySpace users face no shortage of possibilities for representing their music’s genre. Indeed, the fact that they could choose up to three genres from 122 different options means that they had 302,743 different ways to describe their distinctive style. This sort of freedom to represent oneself in so many different ways is certainly resonant of a potentially highly fluid system. However, MySpace musicians stick to a relatively small and recurrent subset of these possibilities, which they combine in highly regular and patterned ways.

This social fact becomes evident through analyzing co-selection of genres as a network, and then examining modularity in that network. This reveals the extent to which genres are likely to be paired with some other genres rather than with others. If such pairings consistently draw from a common pool of MySpace genres, they would not be isolated units combined haphazardly but rather anchored in higher order musical groupings. We shall refer to these complexes as “worlds” and “communities”—islands of musical inbreeding—each of which is structurally distinct from the next. The existence of such higher order groupings would suggest that musicians evidently respect the boundaries of their genres’ communities and worlds, mixing within them rather than between; collective musical norms would evidently structure musicians’ genre choices.

We create a large and complex genre network by mapping the band-provided (self-identified) co-listings of (up to three) genres. Genres are considered “related” once when a band lists them together. In the event that a musician chooses only one genre—thereby providing no information about how genres are associated with one another—the musician’s choices are not included in the analysis. To make sure this did not bias our results, we compared the distribution of genre nominations for genres that are listed alone vs. genres that are listed in some sort of combination. We found that the distributions do not differ substantially. Thus, eliminating single genre selections from our analysis does not bias the genre clusters we write about in any significant way, nor would providing some kind of unique score for single genres change the combinatory patterns that we currently see. In further analyses of the available data it seems that single genre selection has as much to do with incomplete or less active profiles as it does with a full-throated allegiance to a single genre.

For the full population of bands in the data which did list more than one genre designation, Greedy Modularity Optimization is employed to identify genre communities. Greedy Modularity Optimization was developed by Clauset, Newman, and Moore ([65]; see also [66]). This algorithm partitions a network by maximizing its *modularity*, a measure that quantifies a network’s community structure by providing a value for every clustering within a given graph. The general idea is to employ a random graph on the same vertex set that does not have any community structure, and compare the edge density of the clusters in the original graph with the edge density of the clusters in the random graph. The greater the difference between the two edge densities, the more community structure the given clustering describes. We use the version operationalized in R’s IGraph package, which outputs the best community structure (structure with the highest modularity score) possible.

But modularity algorithms, like most clustering algorithms, have no universally accepted significance tests. In other words, there is no consensus as to whether a modularity score of .1, .3, or any value indicates a “real” vs. an arbitrary community structure. In certain situations, however, it can be relatively easy to apply statistical techniques that approximate a significance test. While it is unclear how we would define, let alone test, the significance of the entire community structure discovered in this study, it is relatively straightforward to test whether a single identified community is significantly structurally “separate” from the rest of the large network. This can be done with a Wilcoxon rank-sum test, which, applied to this context, assesses whether there is a statistically significant difference between the number of in-edges and out-edges adjacent to members of a given community. If a community has significantly more in-edges than out-edges, the community is considered statistically significant—a relatively unified group of genres with relatively strong boundaries. And if all its constituent communities are significant, it is reasonable to consider an entire community structure statistically significant.

In this study, running the modularity optimization algorithm and significance testing was procedurally united. In order to identify the most specific genre complexes possible, we do not simply run the modularity optimization once. Instead we run the modularity optimization and the rank-sum test in direct succession and progressively until further dividing a community into smaller, more specific groupings no longer yields statistically significant communities. Our results therefore present a community structure in which all identified communities are indivisible into smaller significant communities and are themselves significant at the p <.01 level.

We used modularity-based approach to community detection because our primary interest is to identify areas of density in a graph composed of weighted and undirected edges. Our data are simple: musicians select genres, and genres are considered related when they are co-selected by many musicians. Areas of density therefore represent in a clear and straightforward way groups of genres that are commonly associated with one another across the millions of musicians in our sample. With only 122 genres and millions of musicians, structure comes from edge weights, so the community detection algorithm chosen must be able to work with edge weights. Since our edges are undirected, the community detection algorithm must be chosen accordingly. Modularity-based approaches are a primary example of internal density approaches that operate on weighted, undirected edges [67]; here we use Igraph's implementation of greedy modularity optimization.

While some modularity-based approaches facilitate the detection of overlapping clusters, we have chosen here to identify non-overlapping genre clusters. This is because we are primarily focused on genre classification. We seek to understand how genres may be categorized and the extent to which those categorizations are clear. Future research may fruitfully pursue potential overlaps between genre clusters—not only measuring boundaries' fuzziness/clarity, but also closely investigating those areas of fuzziness. For this purpose, detecting overlapping communities would be useful.

Many researchers have pointed out that modularity optimization presents a resolution problem, where smaller communities and communities varying in size are difficult to detect [68]. As a solution, we have recursively optimized modularity for discovered clusters (as is also done in [69–70]). While we were able to find reasonable criteria for deciding when to stop partitioning a cluster (Wilcoxon rank-sum test), we were unable to solve the problem of recursive partitions being inconsistent with each other (the "Rock" world is partitioned according to a different mathematical standard than the “Niche” world). However, we do not regard this as a significant problem for our analysis. Our clusters represent different musical worlds that operate according to different logics. The criteria for division and boundary creation should therefore be localized to each sub-cluster instead of generalized to the entire graph.

Though modularity-based community detection makes clear analytical sense for our purposes, as a robustness check we tested several other algorithms that can analyze weighted, undirected edges and clusters genres according to network density or closeness (which we see reasonably similar to density). We found that the clustering produced by Greedy Modularity Optimization was robust across algorithms. If we compare how Greedy Modularity Optimization and alternative algorithms classified genres, we find clusterings that ranged from a low of 93% (Walktrap Community Detection) to a high of 96% (Multi-level Modularity Optimization) similar to Greedy Optimization. Alternative algorithms tended to produce clusters that were less balanced in terms of size, where, for instance, a few genres that Greedy Optimization placed into the “Hip-Hop” world would be placed into the larger and more diverse “Niche” cluster. Although a number of algorithms would produce similar findings, we chose Greedy Modularity optimization because it is the most computationally efficient algorithm and its logic reflects in a straightforward way our intention to find densely interconnected areas of the genre network.

### Three Worlds, 16 Communities

**Fig 2:**
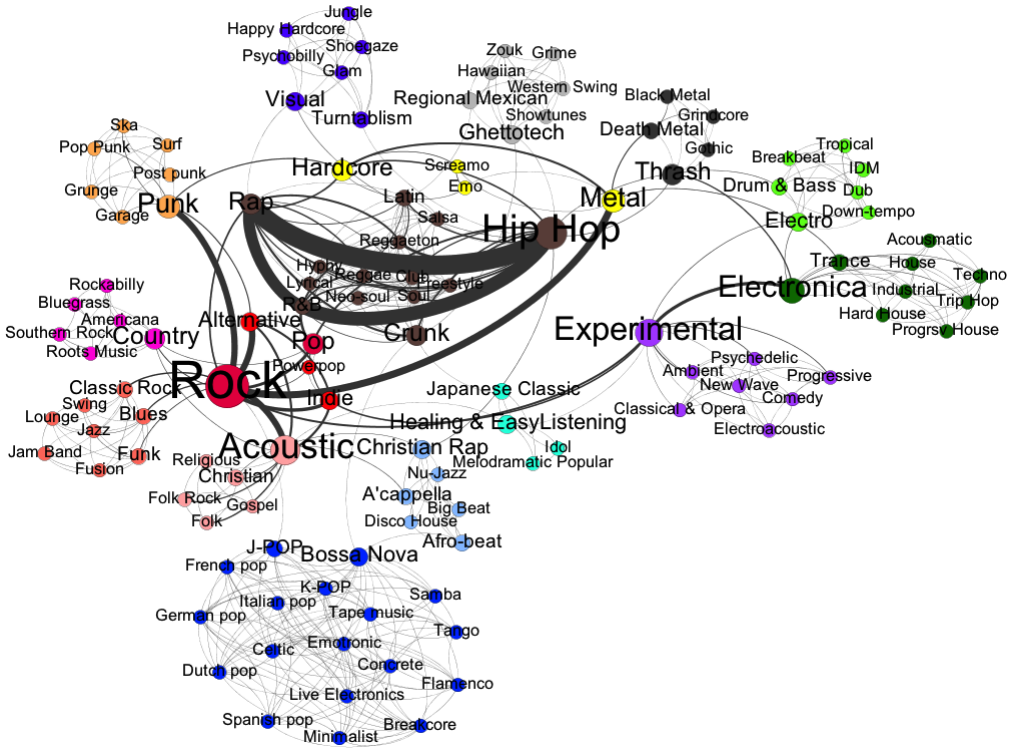
Genres, their Communities, and Interrelations among Communities.

**Fig 3:**
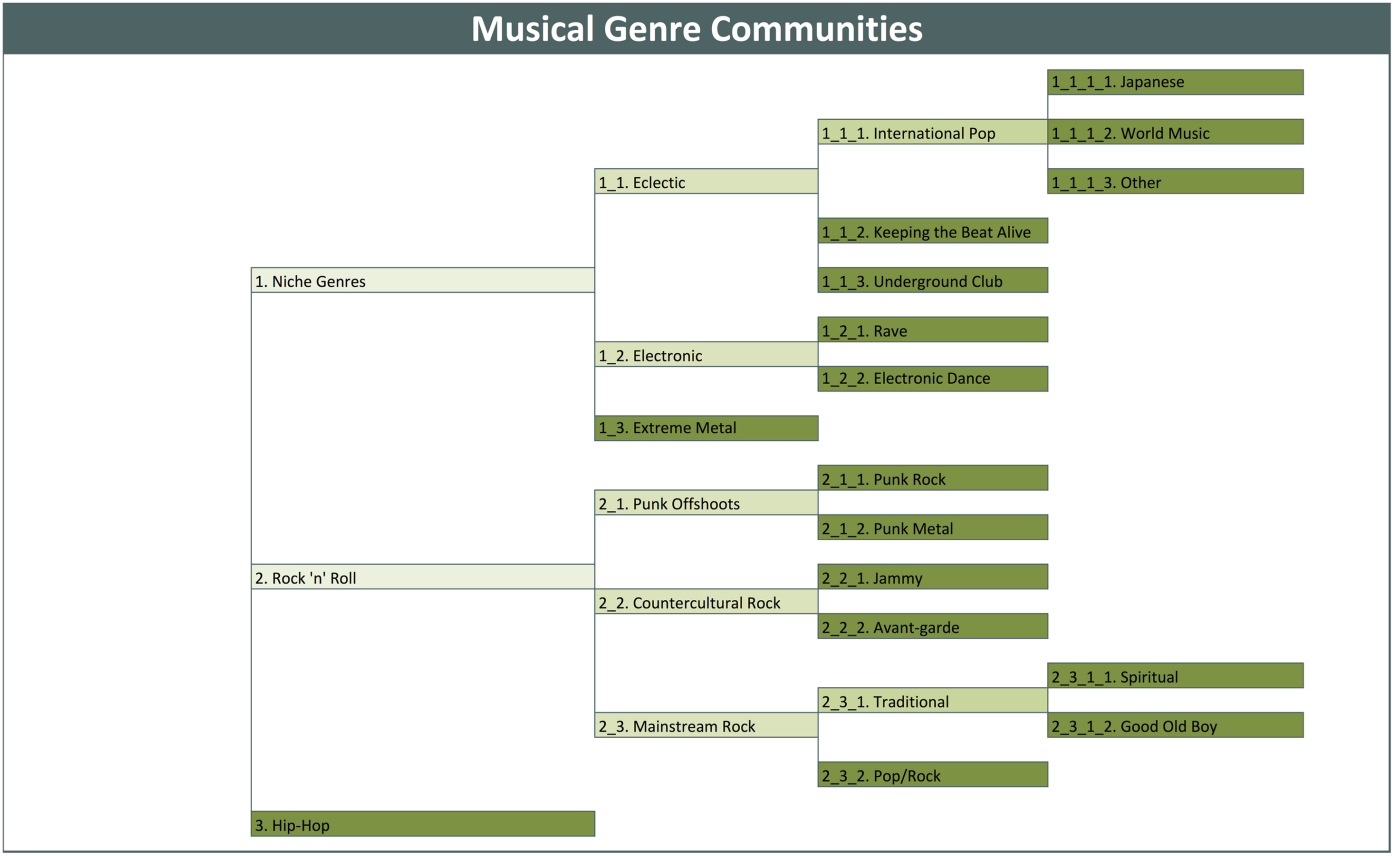
Discovering Musical Genre Communities.

(Fig 2) displays the network of genres in the MySpace universe highlighting how they cluster into genre communities. As modularity in a complex network is difficult to display in a simple visual, (Fig 2) is a modified version of the network. It was created by plotting each of the clusters and its three strongest out-edges. Genre communities are defined by node color, where “warm” colors (red, orange, yellow, pink) represent Rock ‘n’ Roll genres [henceforth referred to as the broad “Rock” world], “cold” colors (blues, greens, purples, grays) represent Niche or Underground genres [henceforth referred to as the broad “Niche” world], and brown represents traditionally African-American and Latino Hip-Hop and Latin-based genres [henceforth referred to as the broad “Hip-Hop” world]. Edge width represents the frequency with which genres are co-chosen. (Fig 3) is a dendrogram that details how a first-order clustering into Rock, Hip-Hop and Niche music worlds is broken down into 16 genre communities and the modularity coefficients at each division. For every division, the community’s in-edges outnumber its out-edges at a statistically significant level (p <.01). Table 2 places each of the 122 genres available on MySpace into 1 of 16 genre communities (see also Figs 2 and 3).

**Table 2:** Membership in Genre Communities.

Genres	Communities
**“Hip-Hop/Rap”**	Club; Crunk; Freestyle; Hip Hop; Hyphy; Latin; Lyrical; Neo-soul; R&B
**“Avant-garde”**	Ambient; Classical & Opera; Comedy; Experimental; Electroacoustic; New Wave; Progressive; Psychedelic
**“Electro/Dance”**	Breakbeat; Downtempo; Drum & Bass; Dub; Electro; IDM; Tropical
**“Extreme Metal”**	Black Metal; Death Metal; Gothic; Grindcore; Thrash
**“Good Old Boy”**	Americana; Bluegrass; Country; Rockabilly; Roots Music; Southern Rock
**“Jammy”**	Blues; Classic Rock; Funk; Fusion; Jam Band; Jazz; Lounge; Swing
**“Japanese”**	Healing & Easy Listening; Idol; Japanese Classic; Melodramatic Popular
**“Keeping the Beat Alive”**	A’Cappella; Afro-beat; Big beat; Christian Rap; Disco House; Nu-Jazz
**“Other”**	Ghettotech; Grime; Hawaiian; Regional Mexican; Showtunes; Western Swing; Zouk
**“Pop/Rock”**	Pop; Powerpop; Rock; Alternative; Indie
**“Punk Metal”**	Emo; Screamo; Hardcore; Metal
**“Punk Rock”**	Garage; Grunge; Pop Punk; Punk; Ska; Surf
**“Rave”**	Acousmatic; Electronica; Hard House; House; Industrial; Techno; Progressive House; Trance; Trip Hop
**“Spiritual”**	Acoustic; Folk; Folk Rock; Christian; Gospel; Religious
**“Underground Club**	Glam; Happy Hardcore; Jungle; Psychobilly; Shoegaze; Turntablism; Visual
**“World Music”**	Bossa Nova; Breakcore; Celtic; Concrete; Dutch Pop; Emotronic; Flamenco; French Pop; German Pop; Italian Pop; J-Pop; K-Pop; Live Electronics; Minimalist; Samba Spanish Pop; Tango; Tape Music

The graph in (Fig 2) indicates how some communities overlap more than others (e.g Pop/Rock and Homegrown American vs. Punk Rock and Rave), and how a few genres—Rock, Hip Hop, Acoustic, Experimental—do much of the work of binding the disparate elements of the musical universe. It is also visible that “Rock” is the touchstone binding the Rock world, an extremely prevalent triad of Hip-Hop, R&B, and Rap is the touchstone for the Hip-Hop world, and Experimental and Electronica are the closest thing to touchstones for the Niche world, which otherwise has no clear center.

Given the strong and statistically significant modularity illustrated in (Fig 2) and detailed in (Fig 3), the analysis suggests that genre distinctions strongly structure the self-presentation of contemporary popular musicians. This dataset does not allow us to compare the strength of these conventions to the past (where much of the extant literature claims that genre conventions are *weakening* over time), but it does indicate that, at least as of 2007, the boundaries between genre worlds were far from extinction. As much as traditional musical genres may have been rhetorically subdivided time and again, those subdivisions still cohere together, operating within distinct boundaries rather than by way of free mixing of musical styles.

Thus, we can satisfyingly answer our first question about whether there is significant clustering of genres at all and can move on to our second: genre selection on MySpace exhibits substantial structural patterning, and those patterns cohere around three musical worlds, two of which may be decomposed into sub-complexes, or genre communities. We performed a placebo test to ensure that the modularity observed in the MySpace network is not due simply to network density, randomly rewiring the network 1000 times. While the modularity coefficient for the actual MySpace network is 0.31, the average modularity for our 1000 random simulations is only .04. This very dense network exhibits almost no modularity at all when its edges are randomly allocated. Thus, the clustering patterns we observe in the MySpace network are highly unlikely to be due to random chance.

Patterning around three musical worlds is most visible in the dendrogram in (Fig 2). The final “end” communities are in the dark green cells. Progressive modularity clustering moves from the left to right of the dendrogram. Furthest on the left, the first-order breakdown separates out three main categories of popular music—Rock, Hip-Hop, and Niche genres. As we move to the right, the Rock world breaks down to its “Subcultural” varieties, which we call “Countercultural,” “Mainstream,” and “Punk Offshoots,” and then finer categories therein. The Hip-Hop world, dominated by Rap, Hip-Hop, and R&B, breaks down no further into statistically significant communities. They comprise both a major musical world and an “end” community—a fact of great significance, as we will see. The “Niche Genres” world represents a variety of less popular (as defined by the frequency with which they are selected by musicians) genres and communities. It is essentially a category encompassing musics not strongly tied to the two dominant poles of Rock and Hip-Hop. These include most notably Electronic music genres, Dark or “Extreme” Metal, and various underground and World Music genres, which emerge and further subdivide as we move to the right of the chart. The Genre-Community membership table demonstrates the considerable face validity of this clustering technique, though the variability in how sub-divided the different worlds are is an intriguing fact that we examine in more detail, below.

### Strength and Differentiation

Turning to our third question, we now seek to uncover the structural properties that distinguish musical worlds from one another. To do so, we are guided by Table 1. Specifically, we ask: What genre complexes have the most and least permeable boundaries? An effective way to measure permeability is by (1) comparing out-edges that reach across the three primary musical worlds that emerge from our first-order clustering above: Rock, Hip-Hop, and Niche. This indicates the proportion of genre communities’ out-edges that bridge *great distance* in the MySpace universe of musical style. In addition, we measure (2) the proportion of each genre’s adjacent edges that are shared with other genres in its community vs. with genres outside its community: in-edges vs. out-edges, in other words. This measure helps us to unpack what the modularity analysis already indicated, that some musical worlds are more internally variegated than others. Combining (1) and (2) allows us to empirically measure musical worlds in terms of their *boundary strength* and *internal differentiation*, and so to situate them in the typology articulated in Table 1.

### Permeability

**Fig 4:**
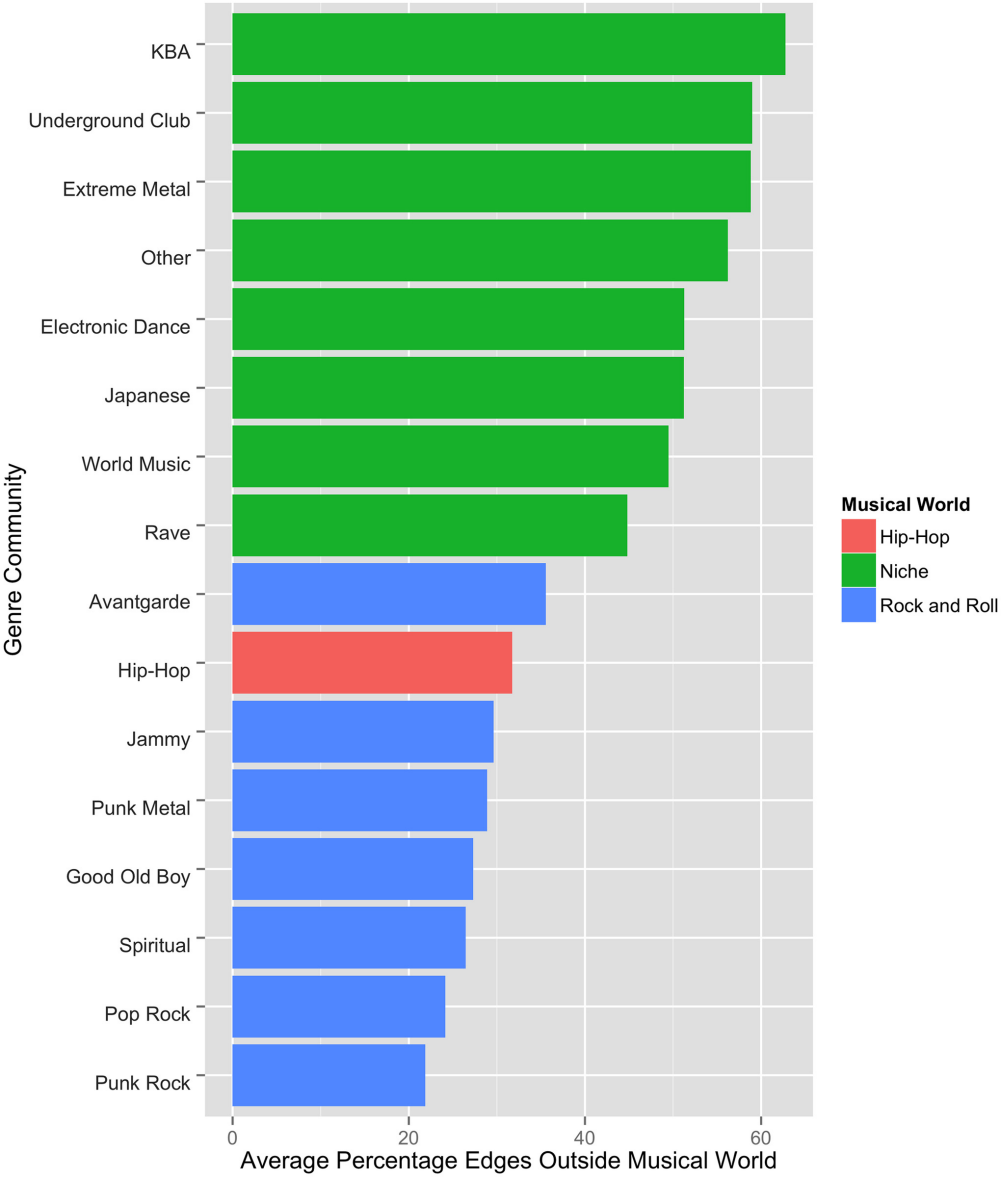
Proportion of Adjacent Edges that Bridge Musical Worlds.

(Fig 4) displays the proportion of each genre community’s edges that are external to its broader musical world. The percentage of extra-world edges for each musical world is listed in Table 3.

**Table 3:** World bridging by musical world.

Musical World	Mean % Edges Outside Musical World
Hip-Hop	31.7
Niche	52.9
Rock	28.0

Niche Genre communities are relatively unbound by their world. Quite often, they reach out to genres outside their world. In fact, ~53% of their edges reach not only outside of a given genre’s home community but also outside of its world to genres in the Rock or Hip-Hop worlds.

By contrast, only ~32% of Hip-Hop bands’ adjacent edges bridge to a different music world. The Rock world is even more impermeable. Only rarely (~28% of the time) do Rock musicians bridge across musical worlds. The worlds of Rock and Hip-Hop thus exhibit *strong* boundaries; the Niche world has relatively *weak* boundaries.

### Differentiation

**Fig 5:**
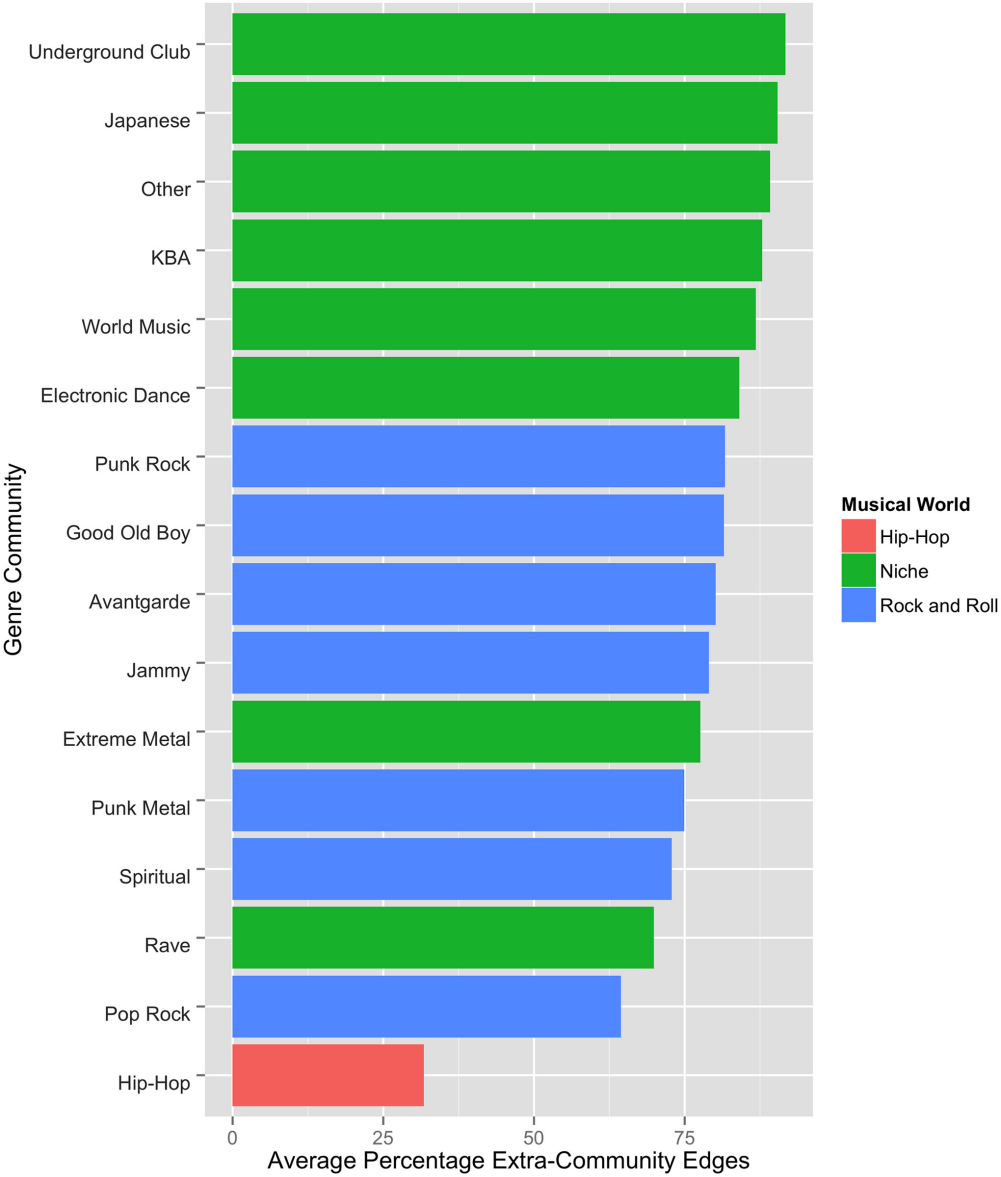
Proportion of Adjacent Edges that Bridge Communities.

(Fig 5) turns our attention to the *internal variegation* of these worlds. The modularity analysis already indicated the key point: Rock and Niche worlds are internally differentiated; Hip-Hop is not. (Fig 5) helps to articulate in more detail how finer-grained genre communities are associated with other communities in their own world and to communities in other musical worlds—the extent to which these differentiated genre communities mix and intermingle. (Fig 5) shows the proportion of each genre community’s edges that are external to that community. The percentage of extra-community edges associated with each broad musical world is listed below it, in Table 4.

**Table 4:** Genre community bridging by musical world.

Musical World	Mean % Edges Outside Community
Hip-Hop	N/A
Rock	77.1
Niche	84.5

On the whole, Niche communities have the most porous boundaries. On average, ~84.5% of edges adjacent to Niche genres bridge across genre communities. Rock music also exhibits substantial inter-penetration, with over 77% of its edges bridging communities. Since the Hip-Hop community is also a world, its community-bridging rate is not comparable to that of the Rock and Niche worlds. Hip-hop’s community-bridging rate is identical to its world-bridging rate of ~31.7%.

### Structural Properties of Musical Worlds

Table 5 synthesizes all of these results. It returns to the typology articulated in Table 1, but now situates the musical worlds within that typology on the basis of our analysis.

**Table 5:** Structural properties of musical worlds.

	High Differentiation	Low Differentiation
**High Boundary Strength**	*Rock Complex*. Multi-centered: bounded subcultural interpenetration	*Hip-Hop Complex*. Single-centered: bounded fluidity
**Low Boundary Strength**	*Niche Complex*. Uncentered: unbound subcultural mixing	Free interchangeability: unbound fluidity

The Rock world is a complex of multiple interpenetrating sub-communities, surrounded by a strong external boundary. Genre mixing across these sub-communities is common; genre mixing beyond the limit of the Rock world is rare. Punk Rock musicians, for example, are more likely to describe themselves through Punk Rock and some non-Punk Rock genre (e.g. Pop Punk and Indie) instead of through Punk-based genres alone (e.g. Pop Punk and Grunge). But they rarely use non-Rock genres to describe themselves. Hip-Hop, however, largely resides in its own musical world, where Hip-Hop bands are extremely likely to describe their music with only other Hip-Hop genres; they are, like Rock musicians, rather unlikely to transcend their musical world. The line between Hip-Hop and not-Hip Hop is strong and rarely crossed. Within its bounds, it is a relatively boundless world, as genres mix fluidly, with little discernible internal sub-cultural differentiation.

The Niche world illustrates low boundary strength and high internal differentiation. Niche genre communities (with notable exceptions of Rave and Extreme Metal music) are the least strictly maintained. Musicians cross them with relative impunity. Obscure Underground Club musicians, for example, are much more likely to define themselves vis-à-vis genres in various other communities (e.g. Happy Hardcore and Electronica) than through a selection of Underground Club genres alone (e.g. Happy Hardcore and Jungle). Niche genre communities are a set of musical sub-cultures only very loosely bound together in a common world, like free-floating solar systems without a strong galactic center. This lack of a center is also visible in (Fig 3), where there is no apparent touchstone binding the disparate Niche communities. They are essentially defined by what they are not, Rock or Hip Hop.

## Conclusion: The Multiple Structural Logics of Popular Music

Drawing from some 3 million bands who have self-classified within a matrix of 122 available genres, we provide the first empirical analysis of this size we are aware of that investigates the structure of popular music by genre. We find that the world of MySpace musicians is not a single world that is made up of “tight” or “loose” genre associations, but instead, is made up of three meso-level genre complexes. A primary result of this paper is to have uncovered the basic properties of these worlds. Rock is a world of sub-cultural differentiation and sub-cultural mixing, operating within a broader common culture of Rock N’ Roll. Sub-cultural formation and transformation produce prevalent interpenetration of sub-cultural identities. The experience of a musician located within such a world is thus one in which musical boundaries are noticeably present, but are flexible and open to constant redefinition and transgression. A heuristic for the generative structural principle at work appears to be: “connect frequently to a common center, but combine some sub-sets more than others.”

Hip-Hop by contrast is a world with a single center but no sub-centers. Surrounded by a strong boundary, Hip-Hop musicians freely combine genres that fall within that boundary. It is a world of structureless fluidity, maintained by a shared connection to a central musical core (composed of Hip-Hop, Rap, and R&B)—a common reference point that over-rides any potential sub-cultural divisions. The experience of a musician located in such a world is of being part of an ever-expanding circle, in which multiple genres may be creatively joined in an egalitarian way as so many faces of a common tradition. Here the generative principle seems to be: “connect anything with anything else, as long as all connect equally to the same center.”

Hip-Hop and Rock ‘n’ Roll are the two major worlds of musical system revealed by the activity of the over 3 million musicians and bands on MySpace.com. Niche genres are the “other” to both: porous communities fluidly combining, lacking any larger touchstone to bind them together.

The worlds of Rock and Hip-Hop do not, however, merely co-exist side-by-side; they are part of the same broader musical system. The boundaries between worlds have a qualitatively different character from the boundaries within a world, for here structural principles collide. To cross over from Rock to Hip-Hop is not simply to engage in more of the same prevalent inter-penetration that characterizes the world of Rock ‘n’ Roll. It is to instead face a basic challenge to the principle of sub-cultural differentiation as a form of musical identity. To cross over from Hip-Hop to Rock is equally challenging. To do so is to potentially abandon the basic principle of egalitarian mixing about a common center in favor of sub-cultural dis-integration. What is differentiation for the one is fragmentation for the other; what is unity for the one is homogeny for the other. The result is that in the popular music system as a whole, the boundary between Rock and Hip-Hop is especially fraught with questions of authenticity and identity.

The complex of multiple creative principles at work among MySpace musicians holds a lesson for the sociology of culture more generally, which in the past decades has been pre-occupied with the rise of a culture of boundary crossing, under the heading of “omnivorousness” [71–73]. However, our analysis indicates that this conceptualization of culture has selective application. It fits the world of Rock ‘n’ Roll well, but it fails to capture the pattern of genre identification and mixing that characterizes the Hip-Hop world, which lacks the sub-cultural structure necessary for bridging to occur but nevertheless exhibits fluid, creative mixing of genres around a common center. The implication, then, is that sociologists of culture would do well to contextualize omnivorousness as one orientation to cultural creativity among others, and to understand how multiple orientations emerge, grow, change, interact, and conflict with one another, in different ways in different places.

This same basic point also suggests that sociologists of culture and scholars in related fields should proceed with caution when making generalizable claims about the ostensible decline or continued importance of genre as a boundary making device. Rather, genre boundaries may simultaneously be highly porous in some complexes and more impenetrable in others. For single or small N case-studies of musical genres, the properties of genre boundaries unearthed may not signal wider trends, but rather the meso-level complexes in which the genre or genres under question operate.

We also suggest that the unearthing of these three meso-level worlds opens up the possibility for more robust longitudinal analysis in future studies. Does the strong core of Rap/hip-hop provide the opportunity space for more unconventional mixing over time, or do the firm boundaries restrict mixing and shed off impure experimentation within the complex? As a niche genre gains in popularity are its genre boundaries more strongly policed, or does it open up space for other popular genres to engage in mixing? Pursuing these questions through alternative data sources—ideally from multiple time periods –would additionally allow us to examine the robustness of the patterns we observe in MySpace as of 2007 to different data collection techniques.

Lastly, rather than treating musical genres as a self-contained relational system, most sociological work examines genre boundaries as they intersect with industry imperatives and audience demand. Based upon our work, more robust analysis of genre complexes and their relationship to both industries and audience is possible. How does musicians’ popularity affect their patterns of genre choices (and vice versa) and do these relationships vary by musical style, place, and available industry resources? We hope to take up these questions in future work.

The major ambition of this paper, however, has not been to examine the antecedents or consequents of popular music’s various genre complexes. It has been to illuminate the structure of those complexes themselves. The discovery of form amidst apparent chaos and parsimonious explanation of complexity are our primary goals. From some 3 million musicians, 122 available genres, and over 300,000 possible combinations, we distill 16 communities nested within 3 worlds at the intersection of 2 dimensions. We lay bare simple heuristics concatenating to form major structural patterns which, for better or worse, continue to govern the classification system that binds and separates popular musicians to and from one another.

## Supporting Information

S1 Table Genres Available on MySpace.com. (DOCX)

## References

[1] Zuckerman EW. The categorical imperative: Securities analysts and the illegitimacy discount. American journal of sociology. 1999 3;104(5):1398–438.

[2] Lena JC, Peterson RA. Classification as culture: Types and trajectories of music genres. American Sociological Review. 2008 73(5):697–718.

[3] Lena JC. Banding together: How communities create genres in popular music. Princeton University Press; 2012 2 12.

[4] Negus K. Music genres and corporate cultures. Routledge; 2013 7 4.

[5] Peterson RA. Creating country music: Fabricating authenticity. University of Chicago Press; 1997 11 24.

[6] Walser R. Running with the devil: Power, gender, and madness in heavy metal music. Wesleyan University Press; 1993 4 1.

[7] Hennion A. The production of success: an anti-musicology of the pop song. Popular Music. 1983 1 1;3:159–93.

[8] Lingo EL, O'Mahony S. Nexus work: Brokerage on creative projects. Administrative Science Quarterly. 2010 3 1;55(1):47–81.

[9] Ahlkvist JA, Faulkner R. “Will this record work for us?”: Managing music formats in commercial radio. Qualitative Sociology. 2002 6 1;25(2):189–215.

[10] Rossman G. Climbing the charts: What radio airplay tells us about the diffusion of innovation. Princeton University Press; 2012.

[11] McLeod K. Genres, subgenres, sub-subgenres and more: Musical and social differentiation within electronic/dance music communities. Journal of Popular Music Studies. 2001 1 1;13(1):59–75.

[12] Van Venrooij A. The aesthetic discourse space of popular music: 1985–86 and 2004–05. Poetics. 2009 8 31;37(4):315–32.

[13] Regev M. The ‘pop-rockization’ of popular music in Hesmondhalgh D, Negus K, editors. Popular music studies. London: Oxford University Press; 2002.

[14] DiMaggio P. Classification in art. American sociological review. 1987 8 1:440–55.

[15] Radway JA. Reading the romance: Women, patriarchy, and popular literature. Univ of North Carolina Press; 1991 11 30.

[16] Beer D. Genre, Boundary Drawing and the Classificatory Imagination. Cultural Sociology. 2013 6 1;7(2):145–60.

[17] Bowker GC, Star SL. Sorting things out: Classification and its consequences. MIT press; 2000 8 25.

[18] Lave J. Cognition in practice: Mind, mathematics and culture in everyday life. Cambridge University Press; 1988 7 29.

[19] Rosch E. Principles of categorization Cognition and categorization, ed. by Rosch Eleanor & Lloyd Barbara B., 27–48.

[20] Zerubavel E. Social mindscapes: An invitation to cognitive sociology. Harvard University Press; 2009 6 1.

[21] Hannan MT, Carroll G. Dynamics of organizational populations: Density, legitimation, and competition. Oxford University Press; 1992.

[22] Haveman HA. Follow the leader: Mimetic isomorphism and entry into new markets. Administrative science quarterly. 1993 12 1:593–627.

[23] Hsu G, Hannan MT. Identities, genres, and organizational forms. Organization Science. 2005 10;16(5):474–90.

[24] Hsu G. Jacks of all trades and masters of none: Audiences' reactions to spanning genres in feature film production. Administrative Science Quarterly. 2006 9 1;51(3):420–50.

[25] Kennedy MT. Getting counted: Markets, media, and reality. American Sociological Review. 2008 4 1;73(2):270–95.

[26] Roy WG, Dowd TJ. What is sociological about music?. Annual Review of Sociology. 2010 8 11;36:183–203.

[27] Blackstone LR. “The Spider Is Alive”: Reassessing Becker's Theory of Artistic Conventions through Southern Italian Music. Symbolic Interaction. 2009 7 1;32(3):184–206.

[28] Burger J. Springsteen on Springsteen: Interviews, Speeches, and Encounters. Chicago Review Press; 2013 4 1.

[29] Carroll GR, Swaminathan A. Why the Microbrewery Movement? Organizational Dynamics of Resource Partitioning in the US Brewing Industry1. American Journal of Sociology. 2000 11;106(3):715–62.

[30] Mezias JM, Mezias SJ. Resource partitioning, the founding of specialist firms, and innovation: The American feature film industry, 1912–1929. Organization Science. 2000 6;11(3):306–22.

[31] Swaminathan A. The proliferation of specialist organizations in the American wine industry, 1941–1990. Administrative Science Quarterly. 1995 12 1:653–80.

[32] Peterson RA, Berger DG. Cycles in symbol production: The case of popular music. American sociological review. 1975 4 1:158–73.

[33] Turow J. Breaking up america. University of Chicago Press; 1997.

[34] Dowd T. From 78s to MP3s: The embedded impact of technology in the market for prerecorded music. The business of culture: Strategic perspectives on entertainment and media. 2006 4 21:205–26.

[35] Katz M. Capturing sound: how technology has changed music. Univ of California Press; 2010.

[36] Leyshon A. The Software Slump?: digital music, the democratisation of technology, and the decline of the recording studio sector within the musical economy. Environment and planning. A. 2009 1 1;41(6):1309.

[37] Sterne J. MP3: The meaning of a format. Duke University Press; 2012 7 17.

[38] Bielby WT, Bielby DD. "All Hits Are Flukes": Institutionalized Decision Making and the Rhetoric of Network Prime-Time Program Development. American Journal of Sociology. 1994 3 1:1287–313.

[39] Carroll GR, Feng M, Le Mens G, McKendrick DG. Organizational evolution with fuzzy technological formats: Tape drive producers in the world market, 1951–1998. Research in the Sociology of Organizations. 2010 1 1;31:203–33.

[40] Leung MD, Sharkey AJ. Out of sight, out of mind? Evidence of perceptual factors in the multiple-category discount. Organization Science. 2013 5 8;25(1):171–84.

[41] Bennett A. Music, Style, and Aging: Growing Old Disgracefully?. Temple University Press; 2013 1 4.

[42] Bennett A, Peterson RA. Music scenes: Local, translocal and virtual. Vanderbilt University Press; 2004.

[43] Clawson MA. When women play the bass: Instrument specialization and gender interpretation in alternative rock music. Gender & society. 1999 4 1;13(2):193–210.

[44] Lena JC. Meaning and membership: samples in rap music, 1979–1995. Poetics. 2004 8 31;32(3):297–310.

[45] Roy WG. Reds, Whites, and Blues: Social Movements, Folk Music, and Race in the United States: Social Movements, Folk Music, and Race in the United States. Princeton University Press; 2010 7 1.

[46] Ennis PH. The seventh stream: The emergence of rocknroll in American popular music. Wesleyan University Press; 1992 12 1.

[47] Kovács B, Hannan MT. The consequences of category spanning depend on contrast. Research in the Sociology of Organizations. 2010 9 13;31:175–201.

[48] Negro G, Hannan MT, Rao H. Categorical contrast and audience appeal: niche width and critical success in winemaking. Industrial and Corporate Change. 2010 10 1;19(5):1397–425.

[49] Hracs BJ. Working in the creative economy: the spatial dynamics of employment risk for musicians in Toronto. PhD dissertation. 2010 University of Toronto.

[50] Caverlee J, Webb S. A Large-Scale Study of MySpace: Observations and Implications for Online Social Networks. InICWSM 2008 Mar 30.

[51] Thelwall M. Social networks, gender, and friending: An analysis of MySpace member profiles. Journal of the American Society for Information Science and Technology. 2008 6 1;59(8):1321–30.

[52] Park N, Kee KF, Valenzuela S. Being immersed in social networking environment: Facebook groups, uses and gratifications, and social outcomes. CyberPsychology & Behavior. 2009 12 1;12(6):729–33. 19619037 10.1089/cpb.2009.0003

[53] Pfeil U, Arjan R, Zaphiris P. Age differences in online social networking–A study of user profiles and the social capital divide among teenagers and older users in MySpace. Computers in Human Behavior. 2009 5 31;25(3):643–54.

[54] Antin J, Earp M. With a little help from my friends: Self-interested and prosocial behavior on MySpace Music. Journal of the American Society for information science and technology. 2010 5 1;61(5):952–63.

[55] Boyle K, Johnson TJ. MySpace is your space? Examining self-presentation of MySpace users. Computers in Human Behavior. 2010 11 30;26(6):1392–9.

[56] Massari L. Analysis of MySpace user profiles. Information Systems Frontiers. 2010 9 1;12(4):361–7.

[57] Moreno MA, Brockman L, Rogers CB, Christakis DA. An evaluation of the distribution of sexual references among “Top 8” MySpace friends. Journal of adolescent health. 2010 10 31;47(4):418–20. doi: 10.1016/j.jadohealth.2010.02.015 20864013 10.1016/j.jadohealth.2010.02.015 PMC2946400

[58] Patchin JW, Hinduja S. Trends in online social networking: Adolescent use of MySpace over time. New Media & Society. 2010 1 19.

[59] Beer D. Making friends with Jarvis Cocker: Music culture in the context of Web 2.0. Cultural Sociology. 2008 7 1;2(2):222–41.

[60] Rothfield L, Coursey D, Lee S, Silver D, Norris W, Hotze T. Chicago music city: A report on the music industry in Chicago. Chicago: Cultural Policy Center at the University of Chicago 2007 Available: https://culturalpolicy.uchicago.edu/sites/culturalpolicy.uchicago.edu/files/CMCFullReport.pdf.

[61] Brydges T, Gilligan S, Matheson Z, Morgan G, Stolarick K. 2013 The geography of Myspace [parts 1, 2, 3] White Papers, Martin Prosperity Institute, Joseph L. Rotman School of Management, University of Toronto.

[62] Hafner K. The well: A story of love, death & real life in the seminal online community. Avalon Publishing Group; 2001 4 1.

[63] Turkle S. Life on the Screen Identity in the Age of the Age of Internet. Touchstone, NY, NY, USA 1995.

[64] Hinduja S, Patchin JW. Personal information of adolescents on the Internet: A quantitative content analysis of MySpace. Journal of Adolescence. 2008 2 29;31(1):125–46. 17604833 10.1016/j.adolescence.2007.05.004

[65] Clauset A, Newman ME, Moore C. Finding community structure in very large networks. Physical review E. 2004 12 6;70(6):066111. 10.1103/PhysRevE.70.066111 15697438

[66] Newman ME. Fast algorithm for detecting community structure in networks. Physical review E. 2004 6 18;69(6):066133. 10.1103/PhysRevE.69.066133 15244693

[67] Coscia M, Giannotti F, Pedreschi D. A Classification for Community Discovery Methods in Complex Networks CoRR, abs/1206.3552, 2012.

[68] Fortunato S. Community detection in graphs CoRR, abs/0906.0612, 2010.

[69] Fortunato S., and Barthelemy M.. Resolution Limit in Community Detection. Proceedings of the National Academy of Sciences. 2007 104, 36. 10.1073/pnas.0605965104 PMC1765466 17190818

[70] Ruan J., Zhang W. Identifying Network Communities with a High Resolution. Phys. Rev. E 2008 77(1), 016104. 10.1103/PhysRevE.77.016104 18351912

[71] Peterson RA. Understanding audience segmentation: From elite and mass to omnivore and univore. Poetics. 1992 8 31;21(4):243–58.

[72] Lizardo O. How cultural tastes shape personal networks. American Sociological Review. 2006 10 1;71(5):778–807.

[73] Lizardo O. Omnivorousness as the bridging of cultural holes: A measurement strategy. Theory and Society. 2014 7 1;43(3–4):395–419.

