# Supplementary material for: Genre Complexes in Popular Music
Source: PLoS One. 2016 May 20;11(5):e0155471. doi: 10.1371/journal.pone.0155471 (PMC4874668; doi:10.1371/journal.pone.0155471)
Supplement: S1 Table — (DOCX) [file pone.0155471.s001.docx]

| **S1 Table: Genres Available on MySpace.com** | | | | |
| --- | --- | --- | --- | --- |
| A'cappella | Disco House | Grindcore | Live Electronics | Rockabilly |
| Acousmatic | Down-tempo | Grunge | Lounge | Roots Music |
| Acoustic | Drum & Bass | Happy Hardcore | Lyrical | Salsa |
| Afro-beat | Dub | Hard House | Melodramatic Popular | Samba |
| Alternative | Dutch pop | Hardcore | Metal | Screamo |
| Ambient | Electro | Hawaiian | Minimalist | Shoegaze |
| Americana | Electroacoustic | Healing&EasyListening | Neo-soul | Showtunes |
| Big Beat | Electronica | Hip Hop | New Wave | Ska |
| Black Metal | Emo | House | Nu-Jazz | Soul |
| Bluegrass | Emotronic | Hyphy | Pop | Southern Rock |
| Blues | Experimental | IDM | Pop Punk | Spanish pop |
| Bossa Nova | Flamenco | Idol | Post punk | Surf |
| Breakbeat | Folk | Indie | Powerpop | Swing |
| Breakcore | Folk Rock | Industrial | Progressive | Tango |
| Celtic | Freestyle | Italian pop | Progrsv House | Tape Music |
| Christian | French pop | J-POP | Psychedelic | Techno |
| Christian Rap | Funk | Jam Band | Psychobilly | Thrash |
| Classic Rock | Fusion | Japanese Classic | Punk | Trance |
| Classical & Opera | Garage | Jazz | R&B | Trip Hop |
| Club | German pop | Jungle | Rap | Tropical |
| Comedy | Ghettotech | K-POP | Reggae | Turntablism |
| Concrete | Glam | Latin | Reggaeton | Visual |
| Country | Gospel |  | Regional Mexican | Western Swing |
| Crunk | Gothic |  | Religious | Zouk |
| Death Metal | Grime |  | Rock |  |
